# Anti-Proliferative Effect of Statins Is Mediated by DNMT1 Inhibition and p21 Expression in OSCC Cells

**DOI:** 10.3390/cancers12082084

**Published:** 2020-07-28

**Authors:** Rachmad Anres Dongoran, Kai-Hung Wang, Tsung-Jen Lin, Ta-Chun Yuan, Chin-Hung Liu

**Affiliations:** 1 Ph.D. Program in Pharmacology and Toxicology, School of Medicine, Tzu Chi University, Hualien 97004, Taiwan; 104721108@gms.tcu.edu.tw (R.A.D.); 104752101@gms.tcu.edu.tw (T.-J.L.); 2 Indonesian Food and Drug Authority (Indonesian FDA), Jakarta 10560, Indonesia; 3 Department of Medical Research, Tzu Chi Hospital, Hualien 97004, Taiwan; kennyhug0201@gmail.com; 4 Department of Life Science, College of Science and Engineering, National Dong Hwa University, Hualien 97401, Taiwan; 5 Department of Pharmacology, School of Medicine, Tzu Chi University, Hualien 97004, Taiwan

**Keywords:** oral squamous cell carcinoma, statins, G_0_/G_1_ cell cycle arrest, p21, DNMT1

## Abstract

Statins, also known as HMG-CoA reductase inhibitors, are a class of cholesterol-lowering drugs and their anti-cancer effects have been studied in different types of malignant diseases. In the present study, we investigated the anti-proliferative effects of statins, including cerivastatin and simvastatin, on oral squamous cell carcinoma (OSCC) cells. Our data showed that statins inhibited the proliferation of three OSCC cell lines in a dose-dependent manner and this growth inhibition was confirmed through G_0_/G_1_ cell cycle arrest. Accordingly, we found the upregulation of p21 and downregulation of cyclin-dependent kinases, including CDK2, CDK4, and CDK6, in the statin-treated cells. Importantly, we clearly showed that statins were able to inhibit the expression of DNA methyltransferase 1 (DNMT1) and further promote the expression of p21. Taken together, our data demonstrated that the anti-proliferative effect of statins is mediated by suppressing DNMT1 expression, thus promoting p21 expression and leading to G_0_/G_1_ cell cycle arrest in OSCC cells.

## 1. Introduction

Oral cancer is one of the most common cancers globally and oral squamous cell carcinoma (OSCC) represents 95% of all forms of it [1,2]. Tobacco use, excessive alcohol consumption, and chronic betel quid chewing are three major risk factors for the development of oral cancer. These factors act separately or synergistically. Other risk factors, such as immune status, human papillomavirus (HPV), family history, pro-inflammatory diet, microbiome, environmental pollutants, occupational exposure, periodontal disease, and type-2 diabetes, have been associated with an increased incidence of oral cancer [3,4]. The therapeutic options, including surgery, radiation therapy, chemotherapy, targeted therapy, and immunotherapy applied in OSCC have expanded in scope and have brought about meaningful improvements in patient outcomes [5]. However, toxicity, side effects, and tumor recurrence following treatment remain major clinical challenges in oral cancer [6]. Therefore, newer drugs and treatments are needed to overcome these challenges.

DNA methylation is one of the epigenetic mechanisms that is involved in OSCC carcinogenesis. DNA methylation, catalyzed by DNA methyltransferase (DNMT), suppresses the gene transcriptional activity resulting from the promoter region being hypermethylated. In OSCC, several important tumor suppressor genes have been reported as being hypermethylated, which is associated with gene silencing [7]. DNMT inhibitor has been approached as the therapeutic target in many cancer treatments, including in OSCC. So far, there are only two FDA-approved DNMT inhibitors for clinical treatment; however, their short lifetime, cytidine deaminase degradation, and toxicity limit their demethylating activity [8]. Therefore, a new generation of DNMT inhibitors is required.

Statins, also known as HMG-CoA (3-hydroxy-3-methylglutaryl-coenzyme A) reductase (HMGCR) inhibitors, are cholesterol-lowering medications that are widely prescribed in the treatment of hypercholesterolemia and cardiovascular diseases. Statins have shown chemoprevention and anticancer effects in a wide range of cancers, such as anaplastic thyroid cancer, liver cancer, bladder cancer, nasopharyngeal cancer, colon cancer, prostate cancer, oral cancer, ovarian cancer, breast cancer, brain cancer, lung cancer, and leukemia. The pleiotropic effects of statins have been observed in several cellular processes, such as cell cycle arrest [9,10,11,12,13,14,15,16,17,18], apoptosis [14,16,18,19,20,21], autophagy [22], migration [18,23], invasion [24], stemness, and metastasis [25]. In addition, the mevalonate pathway (MVP) has been demonstrated as playing an important role in modulating cancer development, because it controls the biosynthesis of cholesterol that is an essential component of cell membranes and as well as other precursors and metabolites [26,27,28]. 

Statins have been reported to inhibit cell proliferation mainly by negative regulation on cell cycle in several cancers. The suppression of cell cycle progression was achieved through the upregulation of cyclin-dependent kinase (CDK) inhibitors, mostly p21 and p27 [9,10,11,13,14,15,17,29,30]. These cell cycle-related tumor suppressor genes (TSGs) bind to cyclin-CDKs complexes and inhibit kinase activity, which leads to the cell cycle arrest [31]. Previous studies have demonstrated several mechanisms of how statins regulate TSGs expression. One study reported the effect of simvastatin on *p21* expression depending on AMPK activation in liver cancer cells [11]. Statins were also found to act as S-phase kinase-associated protein 2 (SKP2) inhibitors in several cancer cells, which resulted in p27 protein accumulation by preventing proteasomal degradation [11,13,14,15]. Interestingly, atorvastatin is able to inhibit DNMT1 and restore the *p16* expression in normal vascular smooth muscle cells through the demethylation of the *p16* promoter region [32]. However, the detailed molecular mechanism of how statins regulate the OSCC cell proliferation remains unclear. More importantly, the ability of statins to act as DNMT inhibitors in cancers has not been investigated yet.

In the present study, we investigated the anticancer effects of statins (cerivastatin and simvastatin) in OSCC cells and their underlying molecular mechanisms. Our data clearly showed that statins inhibited OSCC cell proliferation through G_0_/G_1_ cell cycle arrest, correlating with increased p21, and decreased CDKs expression. Importantly, the treatment of statins suppressed the expression of DNMT1 both in mRNA and protein levels. Together, our data suggested that statins inhibited the expression of DNMT1, causing increased p21 expression and cell cycle arrest in OSCC cells. Thus, statins can serve as therapeutic options for OSCC treatment.

## 2. Results

### 2.1. Statins Inhibited the Proliferation of OSCC Cells

**Figure 1:**
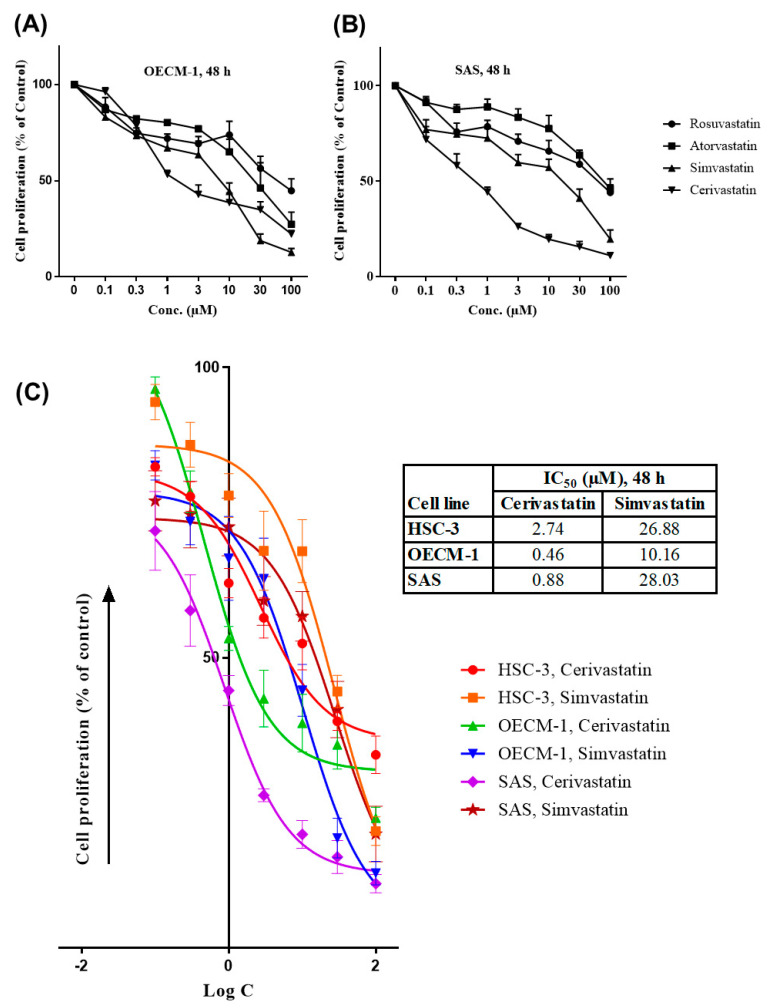
Statins inhibited the proliferation of oral squamous cell carcinoma (OSCC) cells. OSCC cells were treated with various concentrations (0–100 µM) of statins (rosuvastatin, atorvastatin, simvastatin, and cerivastatin) where dimethyl sulfoxide (DMSO) was used as the vehicle control group. The sulforhodamine B (SRB) assay was performed after 48 h of treatment. Cell proliferation of OECM-1 and SAS cells were shown in (**A**,**B**). The half-maximal inhibitory concentration (IC_50_) for three OSCC cell lines, as shown in (**C**), was determined using GraphPad Prism 7 software by plotting nonlinear regression of Log concentration (Log C) of inhibitors (statins) vs. response (cell proliferation, % of control). Error bars represent mean ± SEM from at least 3 independent biological replicates.

We determined the cell proliferation using a sulforhodamine B (SRB) assay after 48 h treatment. The results of the SRB assay were expressed as the percentage of cell proliferation using dimethyl sulfoxide (DMSO) treatment as the vehicle control group. We found that statins inhibited OSCC cell proliferation in a dose-dependent manner, as shown in Figure 1. At first, we tested the anti-proliferation effect of four different statins (rosuvastatin, atorvastatin, simvastatin, and cerivastatin) on OECM-1 and SAS cells. It turned out that simvastatin and cerivastatin had higher growth inhibitory effects than rosuvastatin and atorvastatin (Figure 1A,B). Thereafter, we chose simvastatin and cerivastatin for the subsequent studies. The half-maximal inhibitory concentration (IC_50_) of simvastatin and cerivastatin was also determined in three OSCC cell lines (Figure 1C). Compared to simvastatin, cerivastatin had a lower IC_50_ and exhibited a higher growth inhibitory effect in OSCC cells. Both statins had lower IC_50_ in OECM-1 cells compared to HSC-3 and SAS cells.

### 2.2. Statins Induced G_0_/G_1_ Cell Cycle Arrest and Increased Sub G_1_ Cell Population

**Figure 2:**
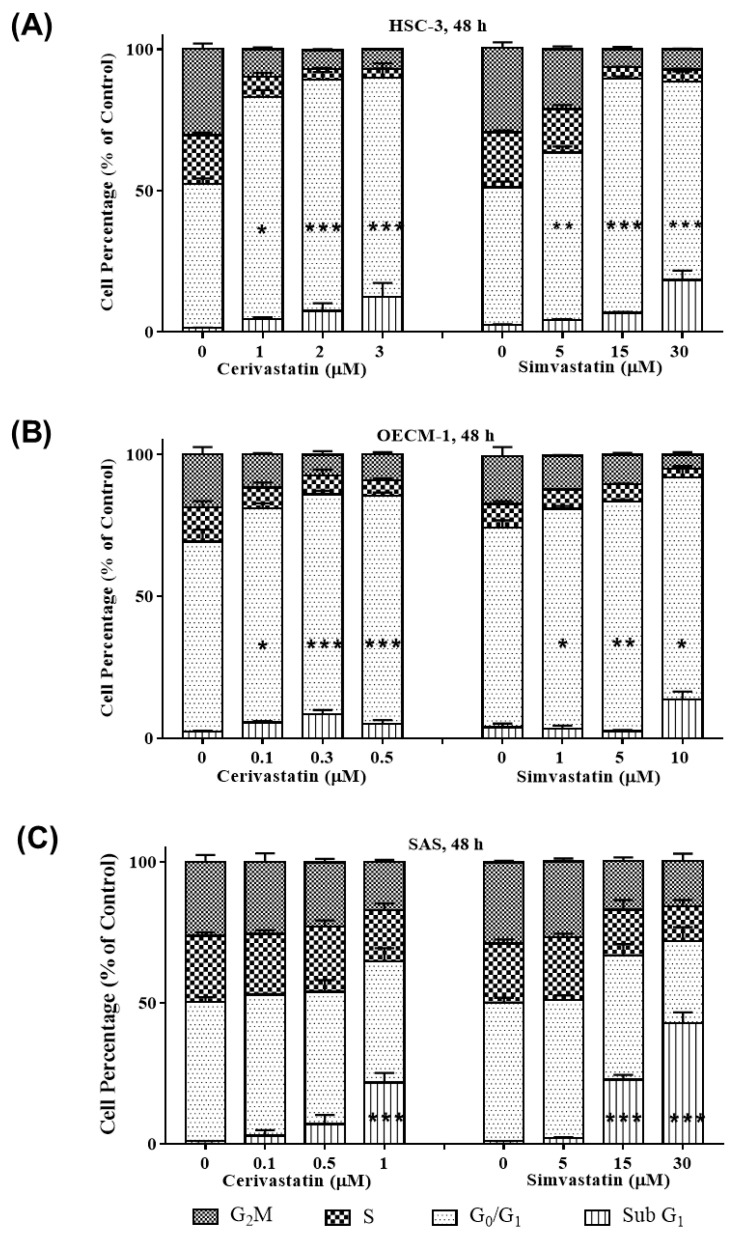
Statins induced G_0_/G_1_ cell cycle arrest and increased Sub G_1_ cell population in OSCC cells. OSCC cells were treated with the indicated concentrations (0-IC_50_) of statins where DMSO was used as the vehicle control group. After 48 h, the percentages of cancer cells in the G_2_/M, S, G_0_/G_1_, and Sub G_1_ phases were analyzed using flow cytometry, as shown for HSC-3 (**A**), OECM-1 (**B**), and SAS (**C**). Error bars represent mean ± SEM from at least 3 independent biological replicates. *p* values were determined using two-way ANOVA and Dunnett’s multiple comparison test (* *p* < 0.05, ** *p* < 0.01, *** *p* < 0.001).

After we observed the inhibitory effect of statins on the proliferation of OSCC cells, we further investigated the mechanism of statin-mediated growth inhibition by analyzing cell cycle distribution. We treated the cells with indicated concentration (0-IC_50_) of statins for 48 h. As shown in Figure S1 and Figure 2, the G_0_/G_1_ cell population of HSC-3 cells increased from 47.61% (control) to 70.67% (cerivastatin, IC_50_, 3 µM), and from 45.91% (control) to 65.96% (simvastatin, IC_50_, 30 µM). For OECM-1, the G_0_/G_1_ cell population increased from 69.99% (control) to 82.62% (cerivastatin, IC_50_, 0.5 µM), and from 66.90% (control) to 86.85% (simvastatin, IC_50_, 10 µM). For SAS, the G_0_/G_1_ cell population slightly increased at the lower dose, but the Sub G_1_ population increased significantly in the higher dose from 0.94% (control) to 28.48% (cerivastatin, IC_50_,1 µM), and from 1.01% (control) to 46.95% (simvastatin, IC_50_, 30 µM). The results collectively suggest that the growth inhibitory effect of statins was associated with the suppression of cell cycle progression in OSCC cells.

### 2.3. Statins Modulated the Expression of p21 and Cyclin-Dependent Kinases (CDKs) 

**Figure 3:**
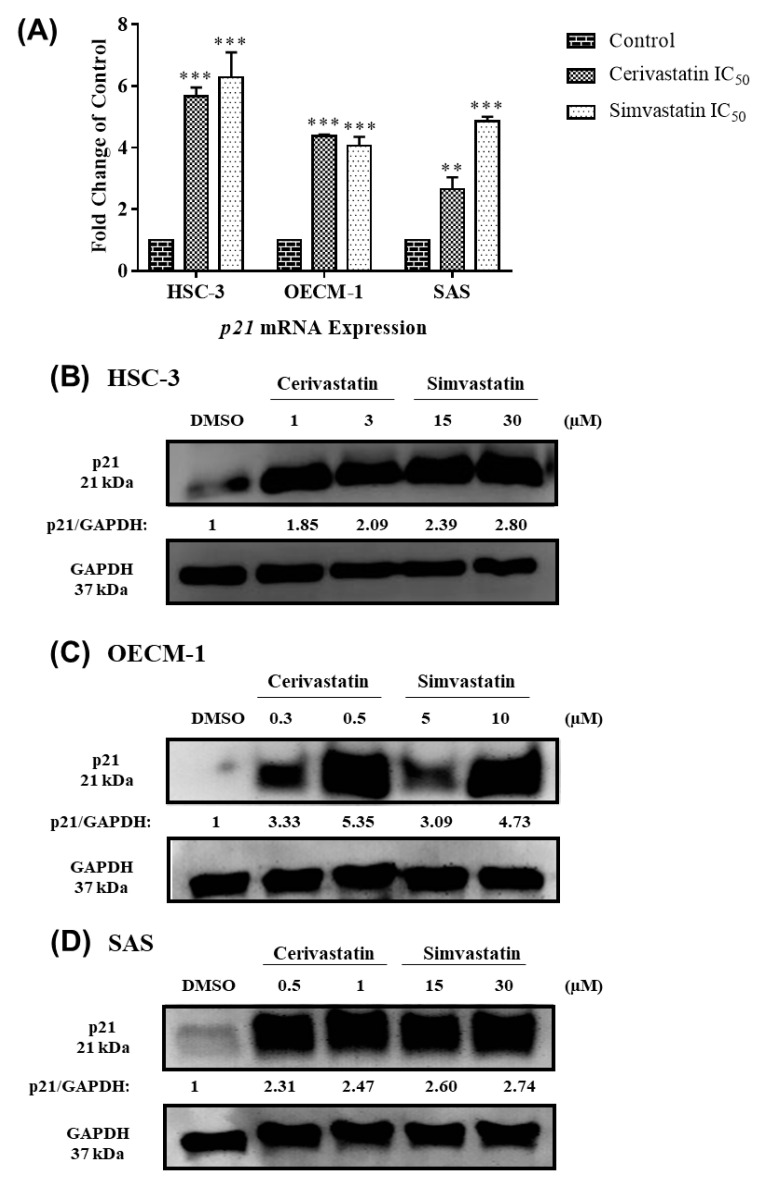
Statins modulated the expression of p21 in OSCC cells. OSCC cells were treated with the indicated concentrations of statins, where DMSO was used as the vehicle control group. After 48 h, the cells were harvested and further processed for RT-qPCR and Western blotting. The *p21* mRNA levels were shown in (**A**). The p21 protein expressions were shown for HSC-3 (**B**), OECM-1 (**C**), and SAS (**D**). Error bars represent mean ± SEM from at least 3 independent biological replicates. *p* values were determined using two ANOVA and Dunnett’s multiple comparison test (** *p* < 0.01, *** *p* < 0.001).

**Figure 4:**
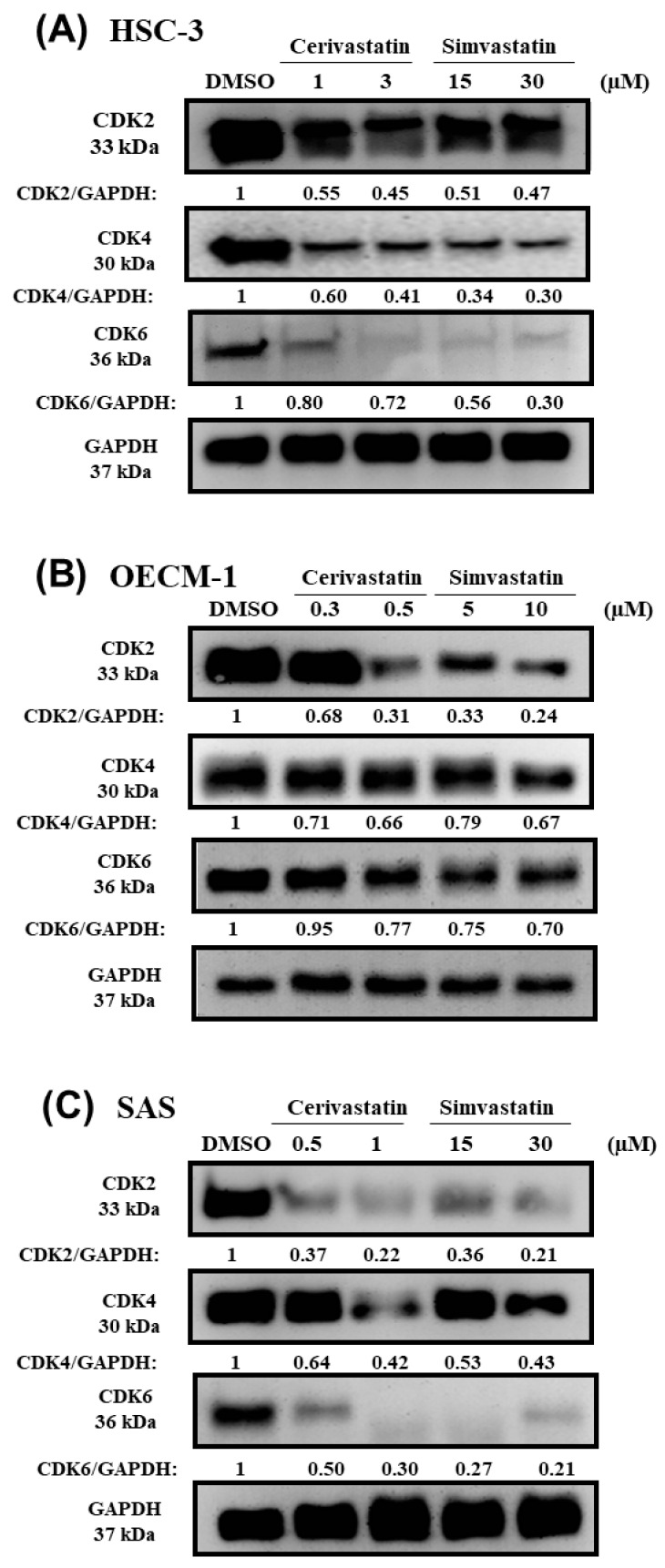
Statins modulated the expression of cyclin-dependent kinases (CDKs) in OSCC cells. OSCC cells were treated with the indicated concentrations of statins where DMSO was used as the vehicle control group. After 48 h, the cells were harvested and further processed for Western blotting. The CDK2, CDK4, and CDK6 protein expressions were shown for HSC-3 (**A**), OECM-1 (**B**), and SAS (**C**).

To examine the molecular mechanism of how statins regulated G_0_/G_1_ cell cycle arrest in OSCC cells, we analyzed the expression of the cell cycle regulatory proteins, including p21, CDK2, CDK4, and CDK6, in the statin-treated OSCC cells. As shown in Figure 3, we found that statins increased *p21* mRNA and protein levels in the three OSCC cell lines. Furthermore, we found that treatment with statins caused the decreased expression of CDK2, CDK4, and CDK6 in OSCC cells (Figure 4A–C). The Western blotting quantification for at least three independent biological replicates is shown for p21 (Figure S2) and CDKs (Figure S3). The data collectively suggested that the statin-induced cell cycle arrest was mediated by modulating the expression of p21 and CDKs in OSCC cells.

### 2.4. Statins Inhibited DNMT1 to Promote p21 Expression

**Figure 5:**
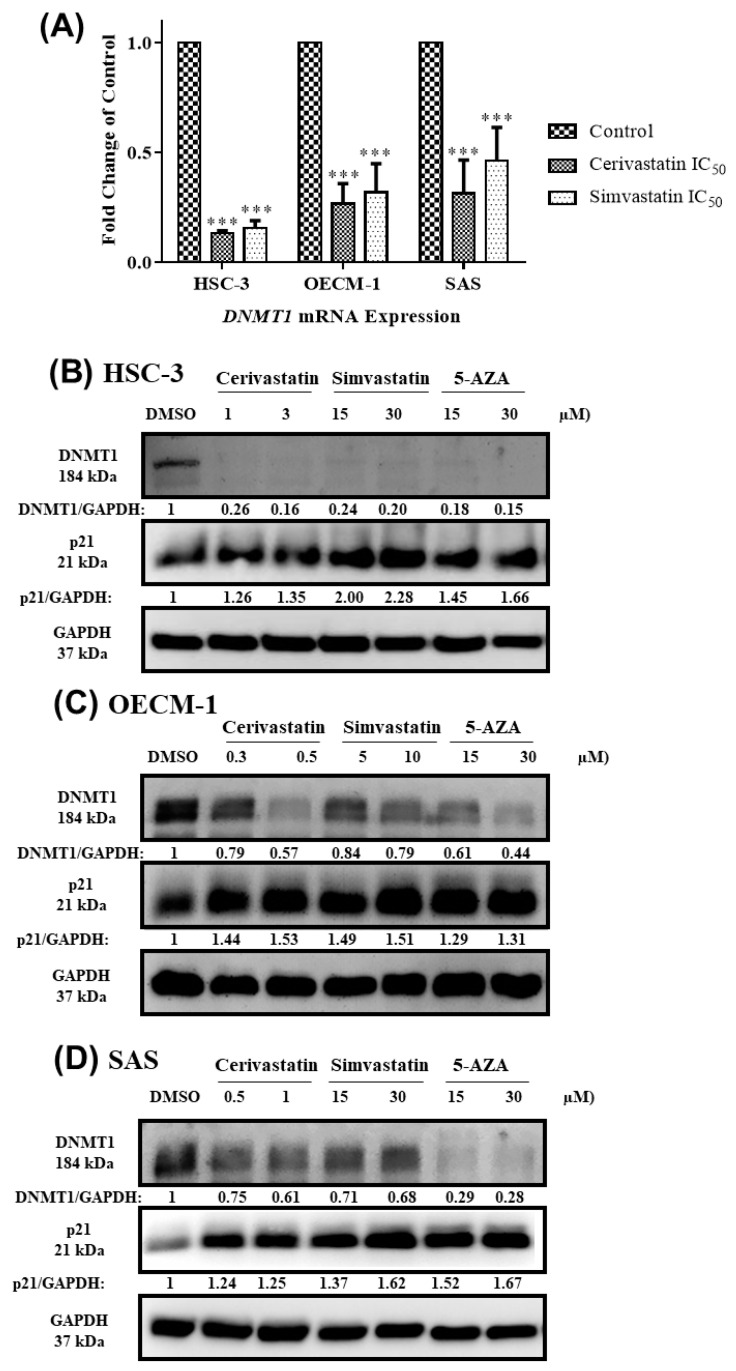
Statins inhibited DNMT1 to promote p21 expression in OSCC cells. OSCC cells were treated with the indicated concentrations of statins where DMSO was used as the vehicle control group. 5-azacytidine (5-AZA) was used as a positive control for DNMT1 inhibition. After 48 h, the cells were harvested and further processed for RT-qPCR and Western blotting. The mRNA levels of *DNMT1* were shown in (**A**). The DNMT1 and p21 protein expressions are shown for HSC-3 (**B**), OECM-1 (**C**), and SAS (**D**). Error bars represent mean ± SEM from at least 3 independent biological replicates. *p* values were determined using two-way ANOVA and Dunnett’s multiple comparison test (*** *p* < 0.001).

It has been reported that hypermethylation on the promoter region of several tumor suppressor genes by DNA methylation leads to transcriptional silencing in OSCC cells [33,34,35,36,37,38,39,40]. Since DNMT1 is responsible for the maintenance of DNA methylation, we further examined whether statins suppressed DNMT1 expression, which caused the transcriptional activation of p21 in OSCC cells. As shown in Figure 5A, the treatment of statins in OSCC cells led to significant decreases in *DNMT1* mRNA levels. Our results further show that statins had an inhibitory effect on the protein levels of DNMT1, which is associated with increased p21 expression. To examine whether statin-modulated p21 expression was mediated by DNMT1 function, we treated cells with 5-azacytidine (5-AZA), an FDA-approved demethylating agent that induced DNMT1 degradation. As shown in Figure 5B–D, treatment of 5-AZA in OSCC cells caused decreases in DNMT1 levels, similar to the results found in statin-treated cells. The Western blotting quantification for at least three independent biological replicates was shown for DNMT1 and p21 (Figure S4). The results suggest that statins could act as DNMT1 inhibitors to promote the transcriptional activation of p21.

## 3. Discussion

**Figure 6:**
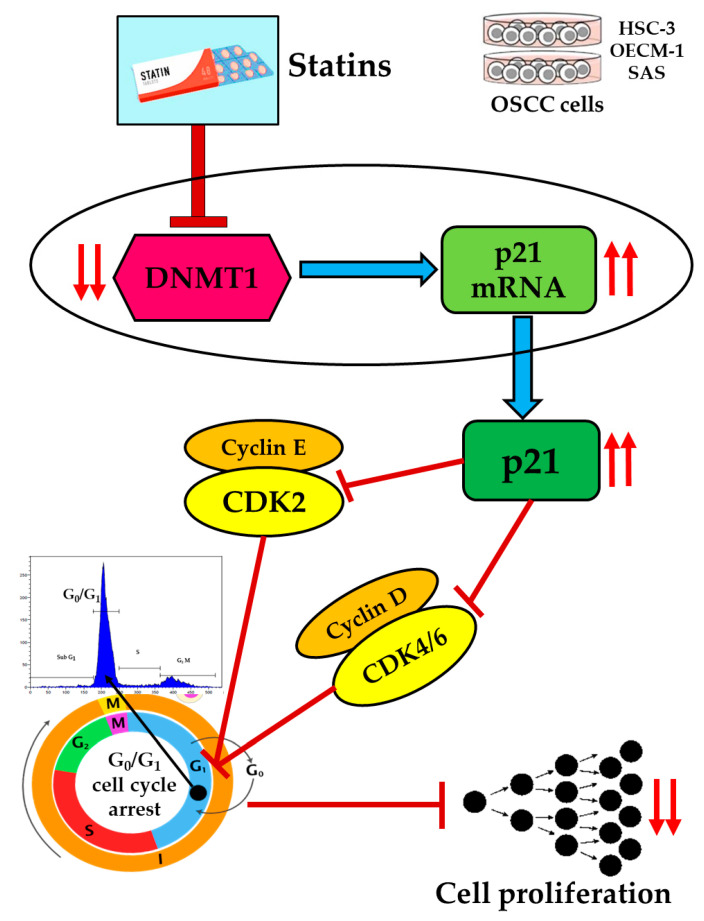
The anti-proliferative effect of statins was mediated by DNMT1 inhibition and p21 expression in OSCC cells. Statins could act as DNMT1 inhibitors and further promoted the upregulation of p21 and downregulation of cyclin-dependent kinases, leading to G_0_/G_1_ cell cycle arrest in OSCC cells.

Accumulated evidence suggested the anti-cancer activity of statins; however, the pharmacological mechanism of statins in regulating cancer cell proliferation is mostly unknown. In this study, we showed that statins had an anti-proliferative effect on OSCC cells, which is due to the G_0_/G_1_ cell cycle arrest. Further studies revealed that statins could function as a DNMT1 inhibitor to activate the transcriptional expression of p21 in OSCC cells, which caused cell cycle arrest and led to reduced cell proliferation (Figure 6). As far as we know, this is the first report showing the anti-proliferative effect of statins by DNMT1 inhibition and p21 expression in OSCC cells. Thus, statins can serve as a therapeutic option for the treatment of patients with OSCC.

We chose cerivastatin and simvastatin for our experiments because they exhibited a higher inhibitory effect on the proliferation of OSCC cells than rosuvastatin and atorvastatin. While cerivastatin is one of the synthetic statins, simvastatin is a natural product-derived statin originally from fungus [41]. Interestingly, both cerivastatin and simvastatin exhibited the same effects and mechanisms in three OSCC cell lines. Cerivastatin had lower IC_50_ with a higher potency on the growth inhibition of OSCC cells. This higher potency of cerivastatin is due to higher lipophilicity compared to simvastatin [42]. Higher lipophilicity of a drug or a molecule is needed to be able to penetrate the lipid bilayer of most cellular membranes. In addition, both cerivastatin and simvastatin had lower IC_50_ in OECM-1 cells, indicating that OECM-1 cells were more sensitive to statin treatment. 

We confirmed that the growth-inhibitory effect of statins in OSCC cells is associated with the suppression of cell cycle progression. Similar to our results, statins have been reported to arrest cancer cells such as G_0_/G_1_ phase arrest in glioma, hypopharyngeal cancer [9], anaplastic thyroid cancer [10], liver cancer [11], and bladder cancer [12]; G_1_ phase in HeLa cells [13], anaplastic thyroid cancer [14], and nasopharyngeal [15]; G_1_S in hepatocellular carcinoma [16] and colon cancer [17]; and S phase in prostate cancer [18]. Moreover, statins upregulated the *p21* mRNA and protein expression and downregulated the expression of CDK2, CDK4, and CDK6 in OSCC cells. These results suggest that the functions of kinases have been inhibited by p21 upregulation. Similar to our results, statins have been reported to upregulate p21 expression in glioma, hypopharyngeal cancer [9], anaplastic thyroid cancer [10,14], liver cancer [11], HeLa cells [13], colon cancer [17], oral cancer [29], and melanoma [30]. p21 also negatively regulates the cell cycle by binding to proliferating cell nuclear antigen (PCNA) through its carboxyl-terminal domain, thus blocking DNA replication [31]. Although the Sub G_1_ cell population was observed increasing significantly in SAS, their *p21* mRNA and protein expression were also upregulated, along with downregulation of CDK2, CDK4, and CDK6. These results indicate that p21 also plays a dominant role in SAS cells. Moreover, studies have shown the pro-apoptotic effect of p21 in different cancers, and its function is mediated by different mechanisms, including caspase-3 activation, p53-dependent activation of p21, modulation pro-apoptotic BAX protein, and Annexin V reactivity [19,29,30,31,43,44,45,46]. Together, statin-induced cell cycle arrest is mediated by modulating the expression of p21 and CDKs in OSCC cells.

Epigenetic modification such as DNA methylation, histone modification, and miRNA regulation have been investigated in OSCC, replacing the concept of cancer as being merely genetic-based alterations. Recently, the promoter region of several important genes was reported as being hypermethylated in OSCC, including *TFP12, SOX17, GATA4, DAPK1, APC, WIF1, RUNX3 E-cadherin, MGMT, hMLH, FLT4, KDR,* and *PTEN,* which leads to their transcriptional silencing and inactivation [33,34,35,36]. In addition, cell cycle-related tumor suppressor genes such as *p14*, *p15*, *p16*, and *RASSF1/2* were reported as being hypermethylated in OSCC cells [37,38,39,40]. Importantly, overexpressed DNMT1 was found both in OSCC cells and clinical specimens [47,48], suggesting the functional role of DNMT1 in OSCC cells. A previous study showed that treatment of 5-AZA-2′-deoxycytidine (5-AZA-CdR) causes a decreased DNMT1 expression and increased p21 expression in colon cancer SW480 cells [49]. Our data clearly show that statins functioned as 5-AZA to suppress DNMT1 expression and promoted p21 expression in OSCC cells. Moreover, previous studies also showed that increased DNMT1 expression mediated by IL-6 stimulation promotes hypermethylation of *p21* and *p53* in lung cancer cells [50], and *SOCS3* in pancreatic cancer cells [51]. More importantly, silencing DNMT1 or 5-AZA-CdR treatment eliminates IL-6-mediated hypermethylation and restores the expression of *p21*, p53, and *SOCS3*. Thus, our data support the notion that the promoter region of the *p21* might have been hypermethylated due to high DNMT1 expression in OSCC cells and treatment with statins, such as 5-AZA, might have eliminated the hypermethylation status of *p21* promoter which led to transcriptional reactivation of *p21* and thus inhibited cell cycle progression in OSCC cells. However, the methylation status of *p21* promoter and the ability of statins to eliminate *p21* hypermethylation through DNMT1 inhibition remains to be elucidated in future studies. So far, no study has reported the hypermethylation status of *p21* in OSCC, but it has been reported in several other cancers such as leukemia, breast cancer, and cervical cancer [52,53,54]. Even so, we clearly showed that statins could act as DNMT1 inhibitors and upregulated p21 expression in OSCC cells.

To our knowledge, only two studies have reported the association between statin treatment and DNMT1 in cancers. By using microarrays, Karlic et al. reported the ability of simvastatin to decrease the mRNA level of DNMT1 in MDA-MB-231 breast cancer cells, PC-3 prostate carcinoma cells, and U2 osteosarcoma cells, but not significantly [55]. In another study, Kodach et al. showed the effect of lovastatin to inhibit DNMT activity in vitro, which leads to *BMP2* promoter demethylation in colorectal cancer cells [56]. In addition, Zhu et al. reported the ability of atorvastatin to inhibit DNMT1 and restore p16 expression in normal vascular smooth muscle cells [32]. Nevertheless, we are the first to provide evidence showing the anti-proliferative effect of statins in OSCC cells by suppressing DNMT1 expression. There are two possible underlying mechanisms of how statins inhibited DNMT1 expression. First, statins inhibited DNMT1 through the inhibition of the mevalonate pathway. Previous studies revealed that blocking the mevalonate pathway using statins subsequently inhibits isoprenylation of the small GTP-binding proteins and therefore the activity of RAS. RAS inhibition further suppresses FLI1 and JNK via RAF signaling into the MAPK pathway. As a consequence, DNMT1 expression is downregulated [57,58,59,60]. Second, statins inhibit DNMT1 expression through the downregulation of IL-6. IL-6 is a robust biomarker in OSCC [61] and statins are well known to suppress IL-6 expression in normal and cancer cells [24,62,63,64,65,66]. Previous studies clearly showed that IL-6 upregulates DNMT1 expression though the IL-6/JAK2/STAT3 pathway [50,51]. Thus, blocking IL-6 induced by statins could suppress DNMT1 expression and subsequently eliminate the hypermethylation process. 

To date, simvastatin has undergone several clinical trials, including a phase II trial in treating patients with stage I-II breast cancer (NCT03454529), a phase I trial in combination with topotecan and cyclophosphamide for CNS tumors (NCT02390843), and a phase II trial in liver cancer (NCT02968810) [67]. Since DNMT1 plays a crucial role in cancer, its inhibitor has been proposed as a therapeutic target in many cancers. By far, 5-AZA and 5-AZA-CdR (decitabine) are the only two FDA-approved DNMT inhibitors for clinical treatment, although their application for cancers is restricted by their relative toxicity and poor chemical stability [8,68]. Recently, another DNMT inhibitor candidate, guadecitabine (by Astex and Otsuka), failed to meet the requirements in a phase III clinical trial for AML patients in 2018 [69]. Therefore, repurposing old and relatively safe drugs such as statins could overcome these clinical and regulatory challenges. In summary, our study clearly showed that statins could act as a DNMT1 inhibitor and further promote p21 expression in OSCC cells. Thus, statins can be used as new and ideal options in oral cancer therapy. 

## 4. Materials and Methods 

### 4.1. Materials

Atorvastatin calcium salt trihydrate (CAS No. 134523038), cerivastatin sodium salt hydrate (CAS No. 143201110), rosuvastatin calcium salt (CAS No. 147098202), simvastatin (CAS No. 79902639), and 5-azacytidine (CAS No. 320672) were purchased from Sigma Aldrich, USA. All drugs dissolved in DMSO (Sigma Aldrich, St. Louis, MO, USA).

### 4.2. Cell Culture and Growth Condition

SAS and HSC-3, human tongue squamous cell carcinoma cells, were originally purchased from the Japanese Collection of Research Bioresources Cell Bank (JCRB, Osaka, Japan). OECM-1, human gingival squamous cell carcinoma cells, were originally derived according to the previous study [70]. SAS and HSC-3 cells were cultured in Dulbecco’s modified Eagle medium (DMEM, Gibco, New York, NY, USA) supplemented with 10% fetal bovine serum (FBS, Gibco, USA) and 1% penicillin-streptomycin (Mediatech, Manassas, VA, USA). OECM-1 cells were cultured in Roswell Park Memorial Institute (RPMI, Gibco, USA) supplemented with 10% fetal bovine serum (FBS, Gibco, USA) and 1% penicillin-streptomycin (Mediatech, USA). All cell lines were cultured at 37 °C in a humidified 5% CO_2_ atmosphere. 

### 4.3. Sulforhodamine B (SRB) Cell Proliferation Assay

Cell proliferation was determined by SRB assay. SRB assay was developed by Skehan and colleagues to investigate cytotoxicity and cell proliferation for anticancer drug screening [71]. OSCC cells were seeded at a density of 5 × 10^3^ cells per well into 96-well plates for 24 h. The cells were then treated with various concentrations of statins (0.1–100 µM) for 48 h, where dimethyl sulfoxide (DMSO, Sigma, St. Louis, MO, USA) was used as the vehicle control group. On the day of the assay, 50 µL trichloroacetic acid (TCA, Sigma, USA) 50% (wt./vol) was gently added to each well directly to the medium supernatant and the plates were incubated at 4 °C for 1 h. The plates were washed four times by submerging the plate in a tub with slow-running tap water and removing excess water by gently tapping the plate into a paper towel. After the last wash, the plates were allowed to air dry at room temperature. Then, 100 µL of sulforhodamine B (SRB, Sigma, USA) 0.4% (wt./vol) solution was added to each well and incubated at room temperature for 1 h, and then the plates were quickly rinsed four times with 200 µL of 1% (vol/vol) acetic acid (Sigma, USA) to remove unbound dye. The plates were allowed to air dry at room temperature. Then, 100 µL of 10 mM Tris base (Amresco, Solon, OH, USA) solution pH 10.5 were added to each well and the plates were shaken on an orbital shaker for 20 min to solubilize the protein-bound dye. The absorbance was measured at 510 nm using the Epoch Microplate Spectrophotometer (Biotek, Taipei, Taiwan). The results of the SRB assay were expressed as the percentage of cell proliferation using dimethyl sulfoxide (DMSO) treatment as the vehicle control group. The half-maximal inhibitory concentration (IC_50_) was determined using GraphPad Prism 7 software by plotting nonlinear regression log concentration (Log C) of inhibitor (statins) vs. response (cell proliferation, % of control).

### 4.4. Flow Cytometry 

Cell cycle distributions were determined by DNA content assay using flow cytometry. OSCC cells were seeded at a density 1 × 10^5^ cells per well into six-well plates. The cells were then treated with the indicated concentration (0-IC_50_ µM) of statins, where dimethyl sulfoxide (DMSO, Sigma, USA) was used as the vehicle control group. After 48 h treatment, the cells were harvested and fixed in 70% ethanol (Merck, Darmstadt, Germany) at −20 °C overnight. On the day of the assay, the cells were washed with phosphate-buffered saline (PBS, MDBio, Taipei, Taiwan) and stained with PBS containing 20 µg/mL propidium iodide (Invitrogen, Carlsbad, CA, USA), octyl phenol ethoxylate 0.1% (J.T. Baker, Phillipsburg, NJ, USA) and RNase A 0.2% (Amresco, USA) for 45 min in the dark at room temperature. The cells populations were analyzed by using Gallios™ Flow Cytometer (Beckman Coulter Life Sciences, Brea, CA, USA). The percentages of cancer cells in the G2/M, S, G_0_/G1, and Sub G1 phases were analyzed using Kaluza software (Beckman Coulter Life Sciences, USA).

### 4.5. RNA Isolation and Quantitative Reverse Transcription-Polymerase Chain Reaction (RT-qPCR) Analysis

The mRNA expression was determined by quantitative reverse transcription-polymerase chain reaction (RT-qPCR). OSCC cells were seeded at a density 1 × 10^6^ cells into a 10 cm petri dish. The cells were then treated with the indicated concentration (IC_50_) of statins, where dimethyl sulfoxide (DMSO, Sigma, USA) was used as the vehicle control group. After 48 h of treatment, total RNA was extracted using the Total RNA Isolation Kit (GeneDirex, Taoyuan, Taiwan) according to the manufacturer’s protocol. The purity and quantity of RNA in each sample was determined using a NanoDrop 2000C Spectrophotometer (Thermo Fisher, Waltham, MA, USA). The GScript First-Strand Synthesis Kit (GeneDirex, Taoyuan, Taiwan) was used to perform reverse transcription from RNA to cDNA according to the manufacturer’s protocol. The primers and annealing temperature of genes analyzed by RT-qPCR are *GAPDH* (F: GAAGGTGAAGGTCGGAGTC, R: GAAGATGGTGATGGGATTTC, 60 °C), *p21^WAF1/CIP1^* (F: GTACCACCCAGCGGACAAGT, R: CCTCATCCCGTGTTCTCCTTT, 60 °C), and *DNMT1* (F: CCGAGTTGGTGATGGTGTGTAC, R: AGGTTGATGTCTGCGTGGTAGC, 60 °C). GAPDH was used as an internal control. The final concentration of cDNA and primers was 100 ng and 900 nM, respectively. Power SYBR Green PCR Master Mix (Thermo Fisher, USA) was used for performing real-time PCR assay. The PCR reaction with 40 cycles (10 m hold at 95 °C, 15 s denature at 95 °C, annealing, and extension 1 m at 60 °C) of amplification was performed using instrument QuantStudio^®^ 5 System (Thermo Fisher, USA) and analyzed using comparative CT (ΔΔCT). Each RT-qPCR was repeated with at least three different RNA and cDNA preparations.

### 4.6. Western Blotting

Protein expression was determined by Western blotting. OSCC cells were seeded at a density of 1 × 10^6^ cells into a 10 cm petri dish. The cells were then treated with the indicated concentration of statins or other treatments, where dimethyl sulfoxide (DMSO, Sigma, USA) was used as the vehicle control group. After 48 h of treatment, cells were harvested and protein was extracted using Pro-Prep^®^ protein extraction solution (Intron Biotechnology, Gyeonggi, Korea) according to the manufacturer’s recommended protocol. Protein concentrations were quantified using a Bio-rad protein assay reagent (Bio-rad, Hercules, CA, USA). NuPAGE™ lithium dodecyl sulfate (Invitrogen, Carlsbad, CA, USA) was used to prepare protein samples for denaturing at 70 °C for 10 min. The protein (20–30 µg per lane) was separated with different gel percentages (8–14%) of sodium dodecyl sulfate-polyacrylamide gel electrophoresis (SDS-PAGE) gel, depending on the size of the protein of interest. The gel was run for 30 min at 70 V and the voltage was increased to 120 V to finish the run in about 90 min. Protein was transferred into an Immobilon^®^-P polyvinylidene difluoride (PVDF) transfer membrane (EMD Millipore, Burlington, MA, USA) by using semidry electroblotting transfer machine (Amersham Biosciences, Little Chalfont, UK) for 1 h at 90 mA. The membrane was blocked for 1 h at room temperature with 5% non-fat milk in phosphate buffered saline with 0.1% Tween 20 (PBST). The primary antibody solution against the target was added and incubated overnight at 4 °C. The primary antibodies, dilution and the host that were used in this study were GAPDH (Invitrogen, 1:5000, mouse), p21^WAF1/CIP1^ (Cell Signaling, 1:1000, rabbit), CDK2 (Cell Signaling, 1:1000, rabbit), CDK4 (Cell Signaling, 1:1000, mouse), CDK6 (Cell Signaling, 1:1000, rabbit), and DNMT1 (Santa Cruz Biotechnology, 1:500, mouse). The membrane was rinsed three times for 10 min with PBST. The rinsed membrane was incubated in the secondary antibody solution for 1 h at room temperature. The secondary antibodies, dilution, and the host that were used in this study were anti-mouse IgG (Cell Signaling, 1:1000, horse) and anti-rabbit IgG (Cell Signaling, 1:1000, goat). The membrane was rinsed again three times for 10 min with PBST. For imaging, Immobilon^®^ Western Chemiluminescent HRP substrate (Millipore, USA) was applied to the blot following the manufacturer’s suggestion. Chemiluminescent signals were captured using a UVP Biospcetrum 810 Imaging System machine (Thermo Fisher, USA). For data analysis, Image J^®^ (National Institute of Health, Bethesda, MD, USA) analysis software was used to read the band intensity of the loading control protein and the target protein. Original images for Western blotting are shown in Figure S5.

## 5. Conclusions

Our study provides clear and new findings of how statins regulated the proliferation of OSCC cells. Two different statins have been investigated and exhibited the same molecular mechanism on three OSCC cell lines. Statins inhibited OSCC cell proliferation through G_0_/G_1_ cell cycle arrest. In accordance, p21 was found to be upregulated, while CDKs were found to be downregulated. More importantly, statins could act as DNMT1 inhibitors, modulate p21 expression, and lead to G_0_/G_1_ cell cycle arrest. Thus, statins represent an ideal candidate for repositioning as DNMT1 inhibitors in OSCC therapy.

## Supplementary Materials

The following are available online at https://www.mdpi.com/2072-6694/12/8/2084/s1, Figure S1: Cell cycle distribution raw data; Figure S2: Western blotting quantification of p21; Figure S3: Western blotting quantification of CDKs; Figure S4: Western blotting quantification of DNMT1 and p21; Figure S5: Original images for Western blotting.
