# Supplementary material for: Anti-Proliferative Effect of Statins Is Mediated by DNMT1 Inhibition and p21 Expression in OSCC Cells
Source: Cancers (Basel). 2020 Jul 28;12(8):2084. doi: 10.3390/cancers12082084 (PMC7463937; doi:10.3390/cancers12082084)
Supplement: Supplementary file 1 [file cancers-12-02084-s001.pdf]

Figure S1. Cell Cycle Distribution Raw Data

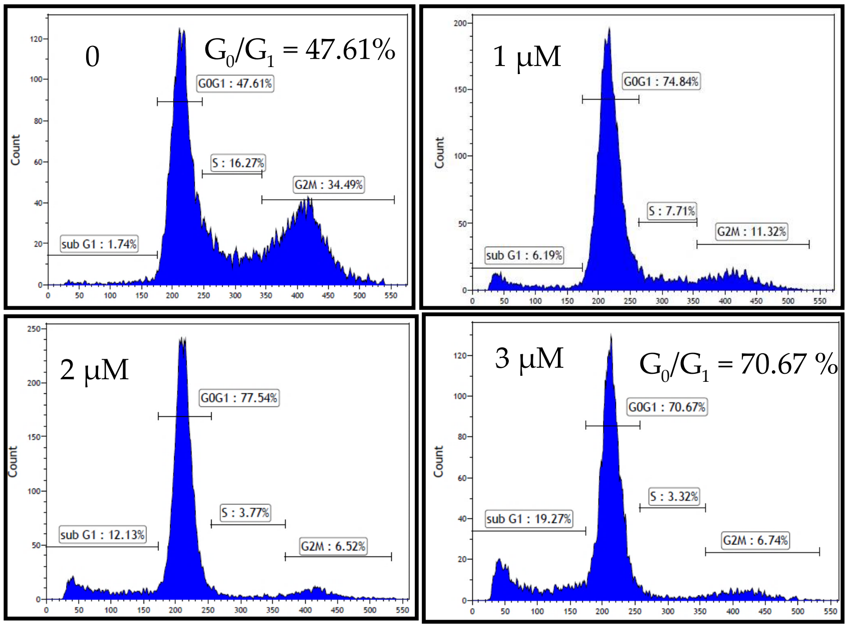

A. HSC-3, Cerivastatin 48 h

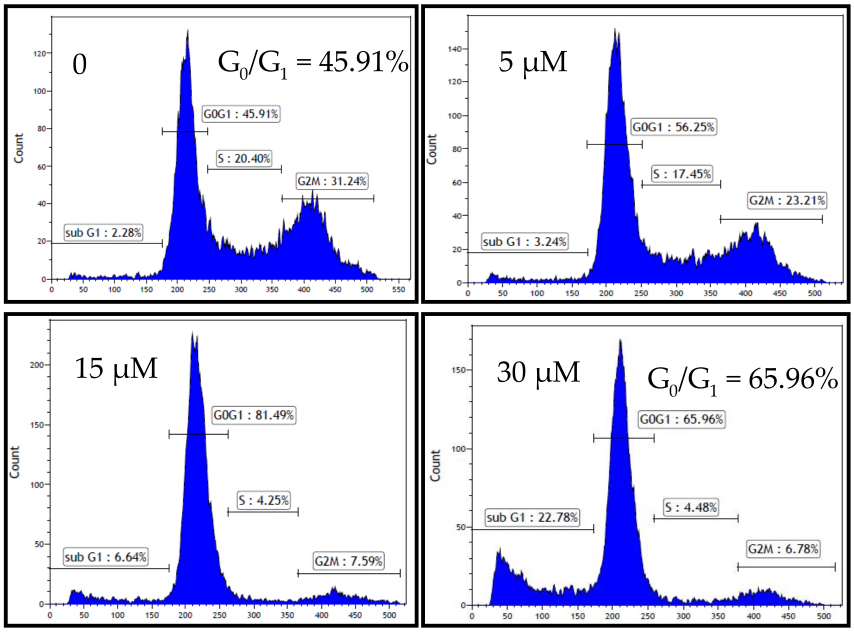

B. HSC-3, Simvastatin 48 h

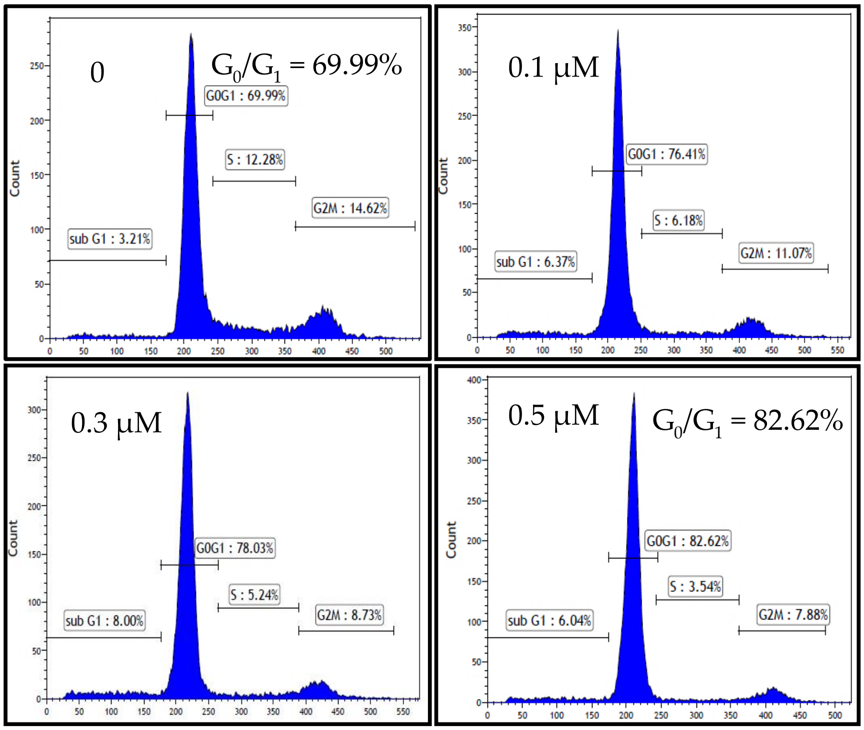

C. OECM-1, Cerivastatin 48 h

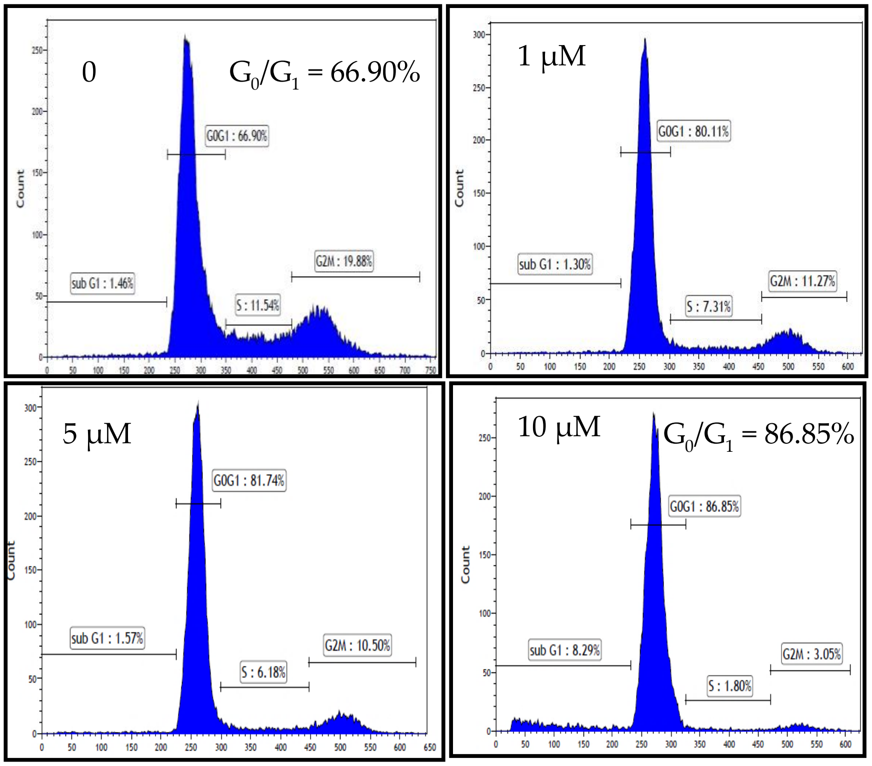

D. OECM-1, Simvastatin 48 h

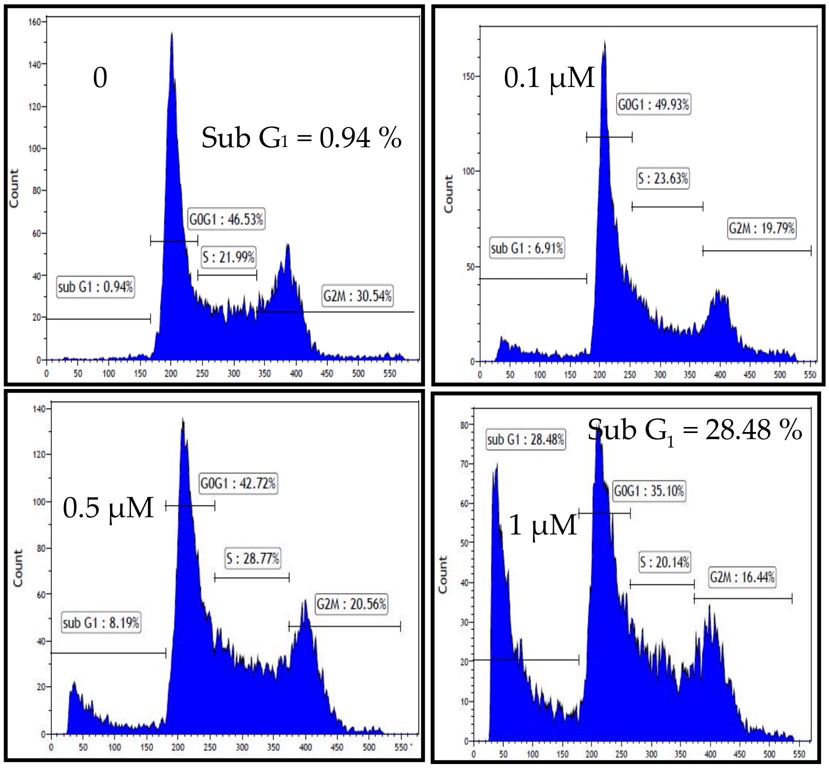

E. SAS, Cerivastatin 48 h

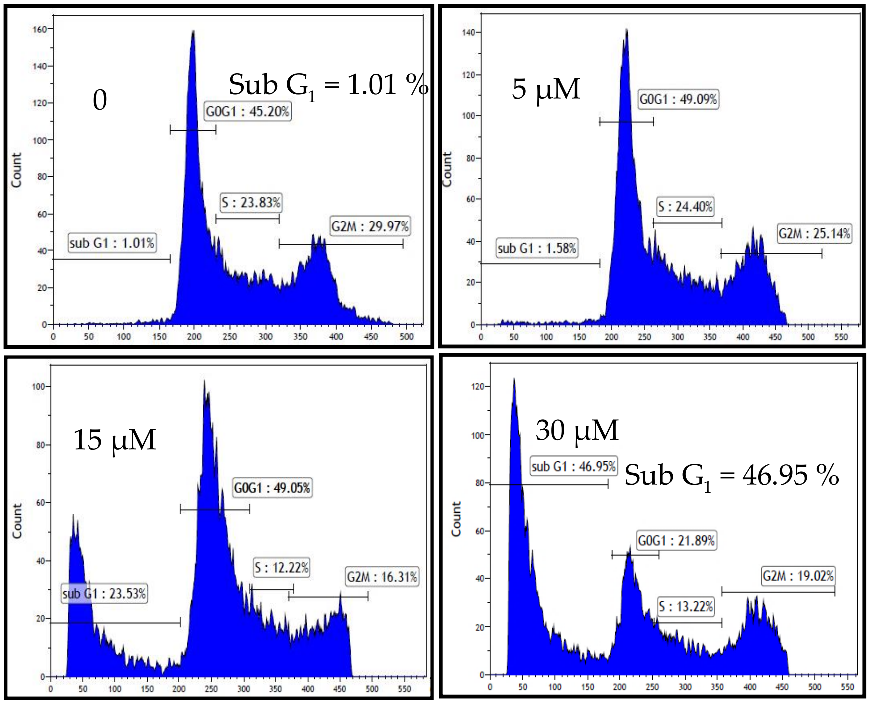

F. SAS, Simvastatin 48 h

Figure S1. Cell cycle distribution raw data. The percentages of cancer cells in the G_2/M, S, G_0/G_1, and Sub G_1 phases were analyzed using flow cytometry as shown for HSC-3 (**A-B**), OECM-1 (**C-D**), and SAS (**E-F**).

Figure S2. Western Blotting Quantification of p21

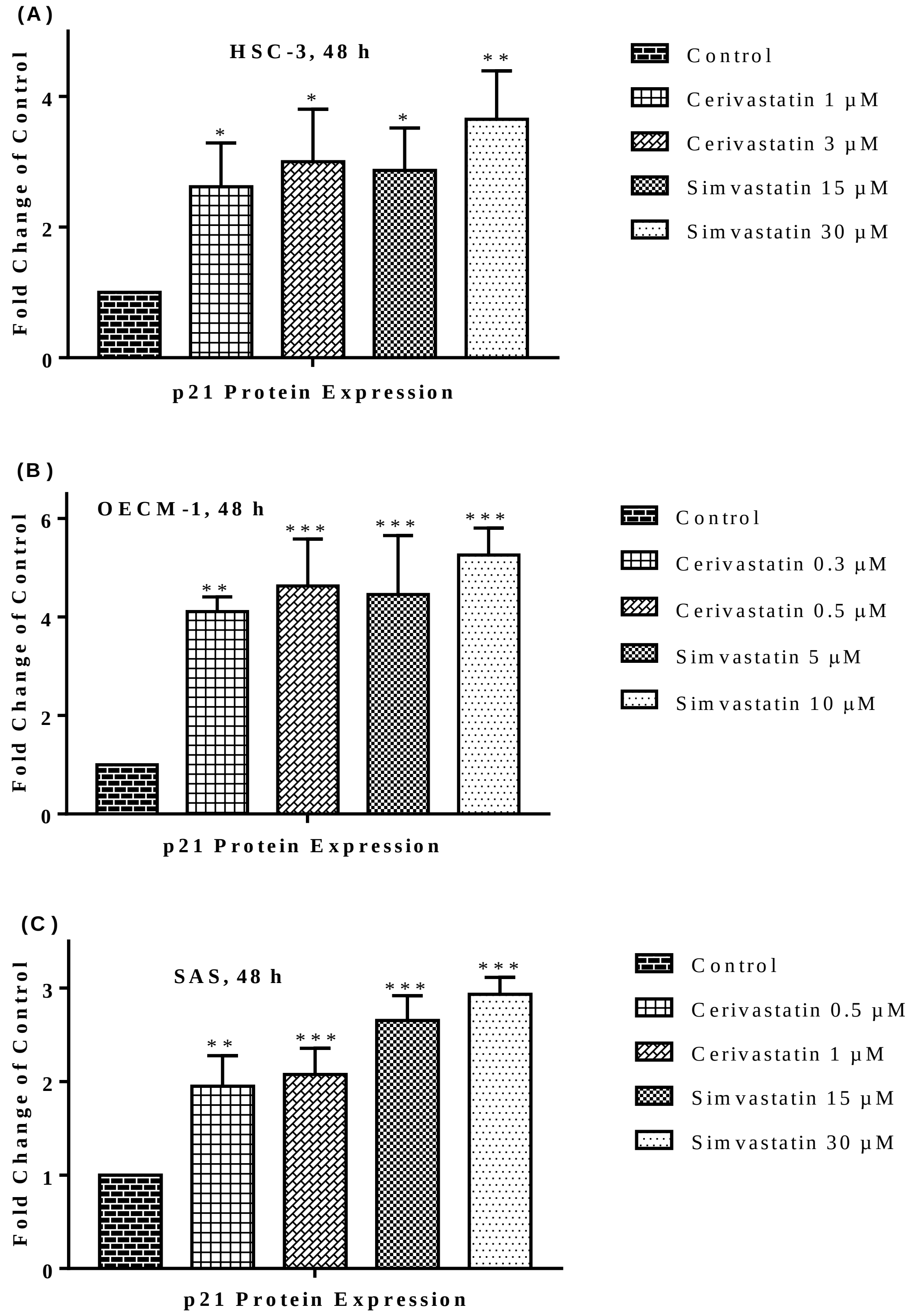

Figure S2. Western blotting quantification of p21. Protein expression of p21 was shown for HSC-3 (**A**), OECM-1 (**B**), and SAS (**C**). Error bars represent mean ± SEM from at least 3 independent biological replicates. P values were determined using one-way ANOVA and Dunnett's multiple comparison test (*P < 0.05, **P < 0.01, ***P < 0.001).

Figure S3. Western Blotting Quantification of CDKs

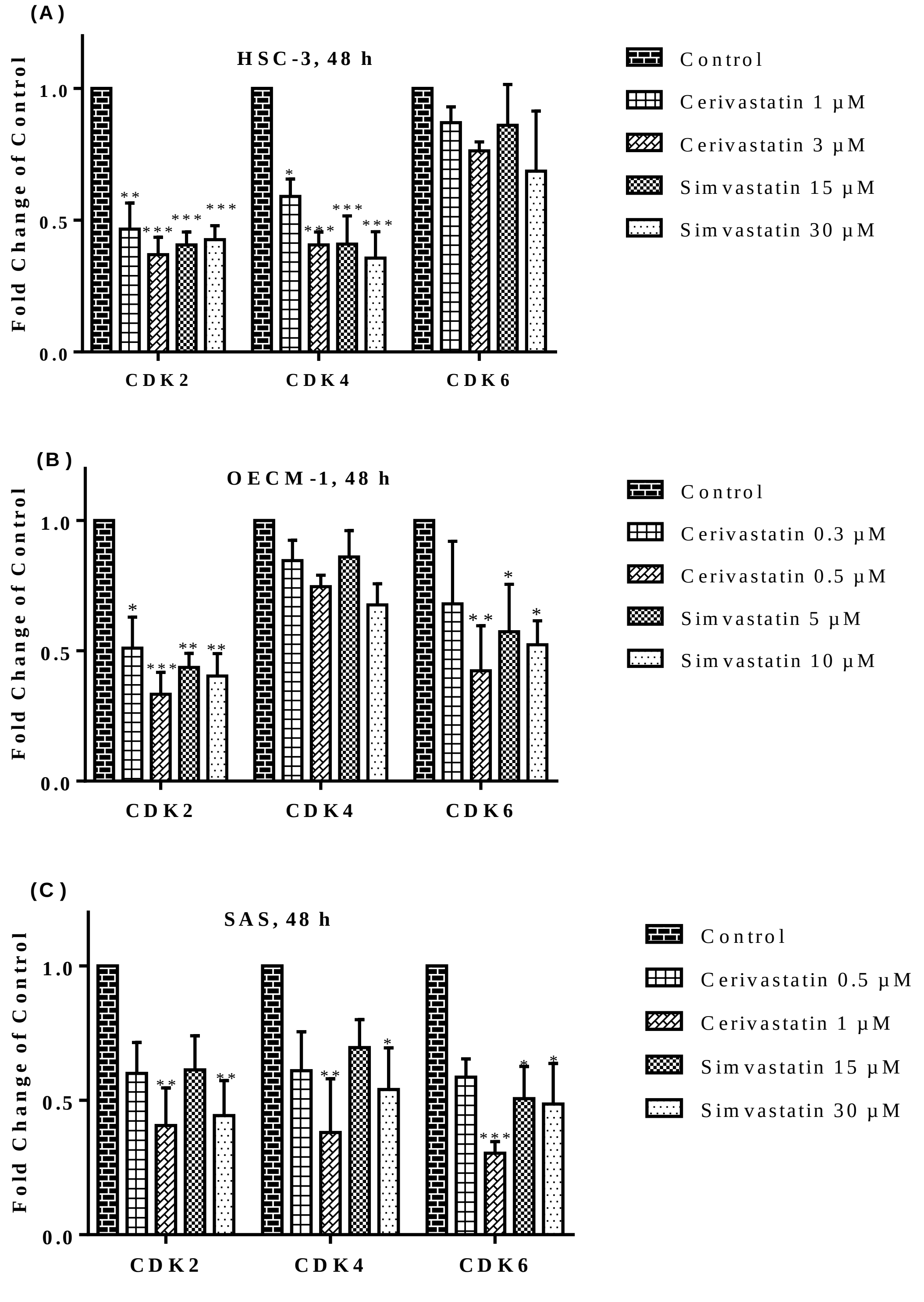

Figure S3. Western blotting quantification of CDKs. Protein expression of CDKs was shown for HSC-3 (**A**), OECM-1 (**B**), and SAS (**C**). Error bars represent mean ± SEM from at least 3 independent biological replicates. *P* values were determined using two-way ANOVA and Dunnett's multiple comparison test ($^{*}P < 0.05$, $^{**}P < 0.01$, $^{***}P < 0.001$).

Figure S4. Western Blotting Quantification of DNMT1 and p21

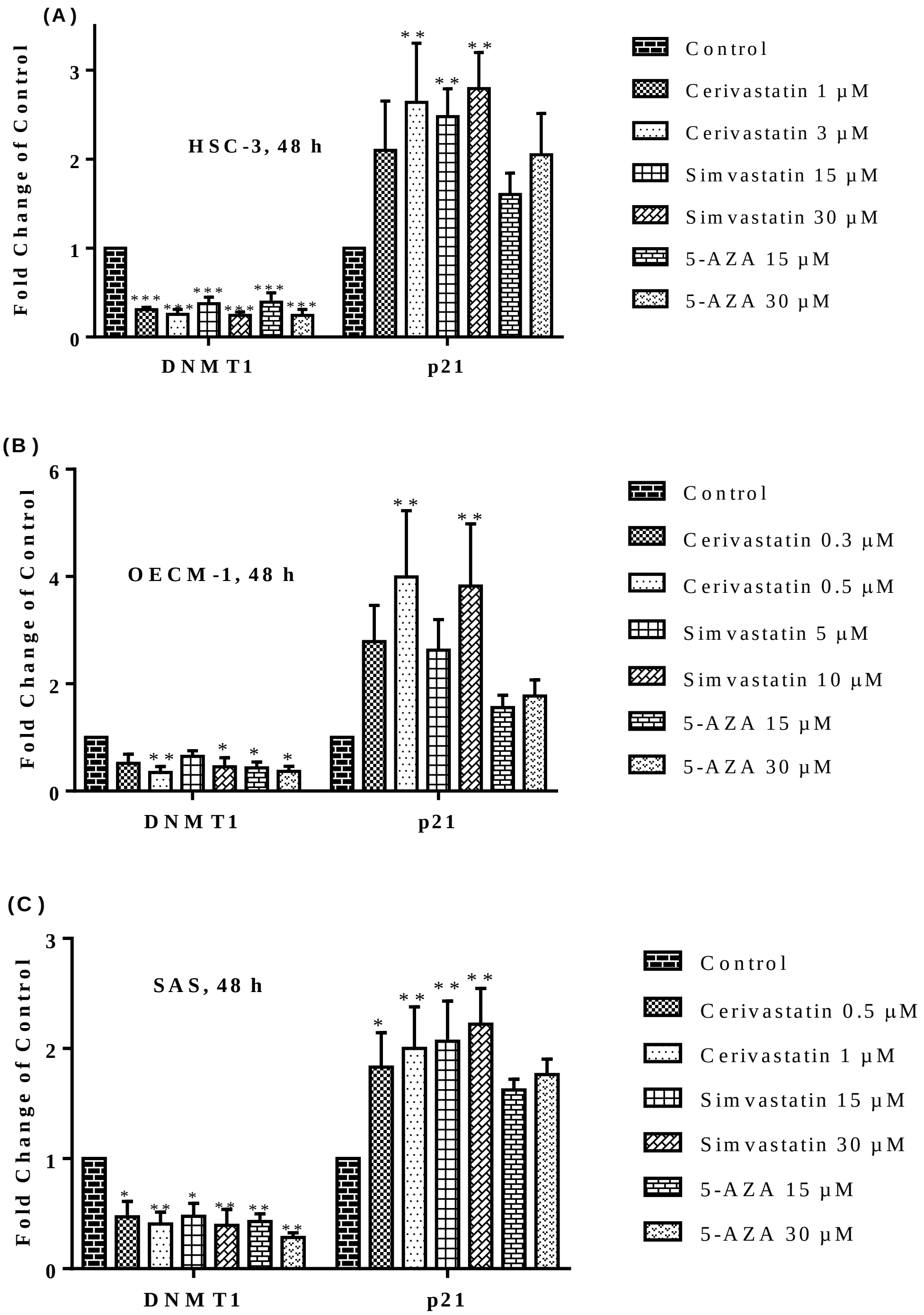

Figure S4. Western blotting quantification of DNMT1 and p21. The protein expression of DNMT1 and p21 was shown for HSC-3 (**A**), OECM-1 (**B**), and SAS (**C**). Error bars represent mean ± SEM from at least 3 independent biological replicates. *P* values were determined using two-way ANOVA and Dunnett's multiple comparison test ($^{*}P < 0.05$, $^{**}P < 0.01$, $^{***}P < 0.001$).

Figure S5. Original Images for Western Blotting

1. **p21 (Figure 3)**

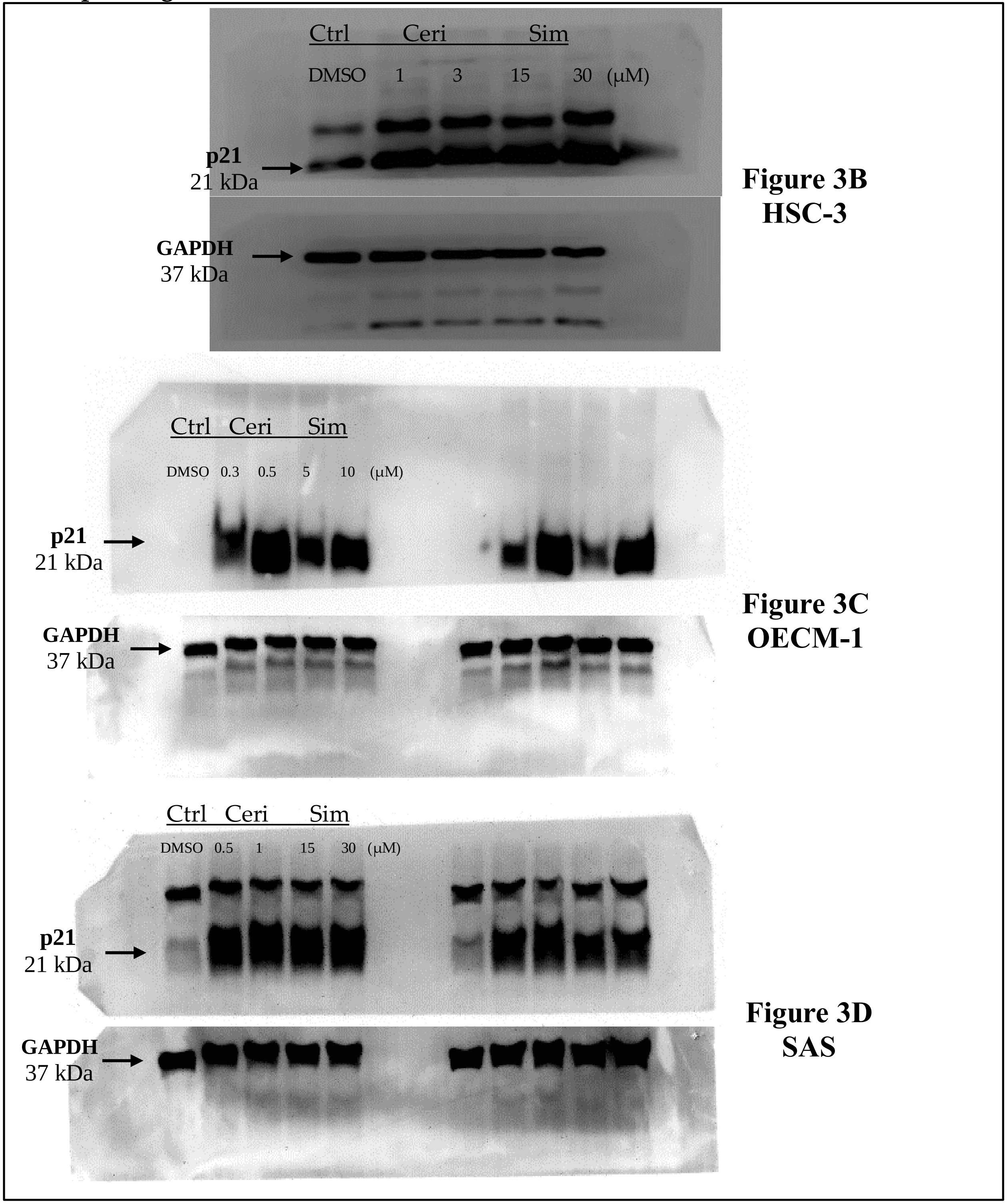

2. CDKs (Figure 4)

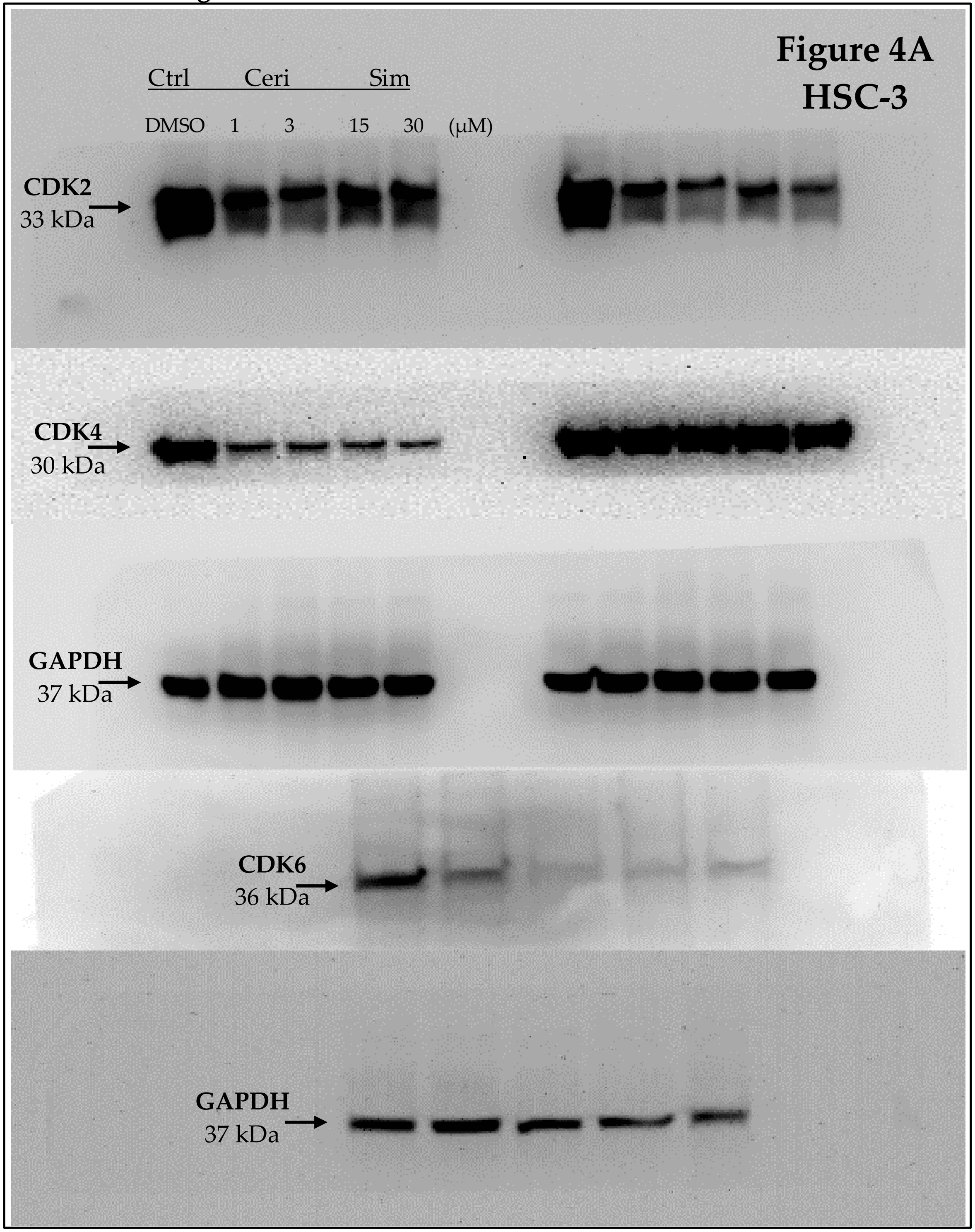

CDKs (Figure 4)…

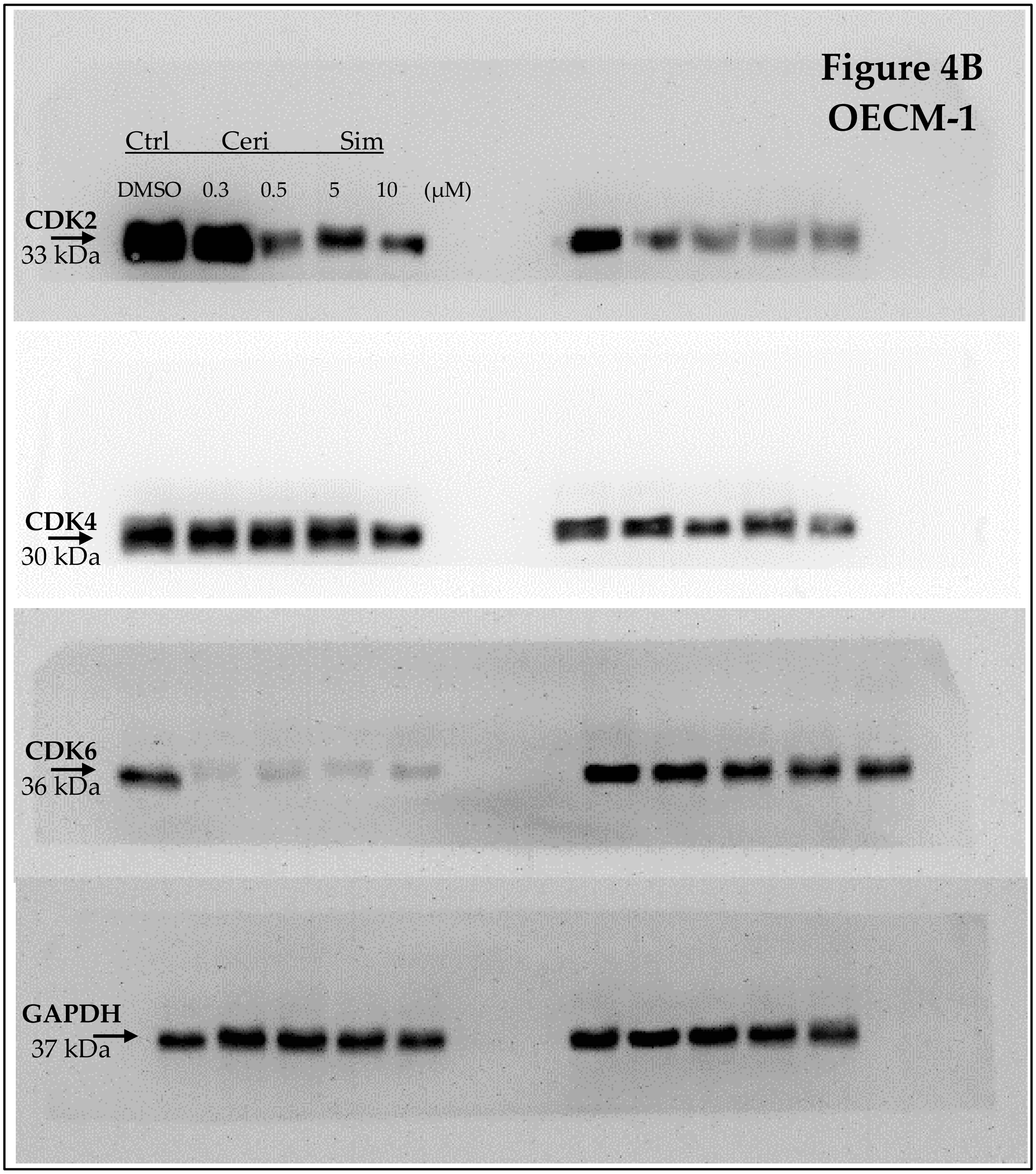

CDKs (Figure 4)…

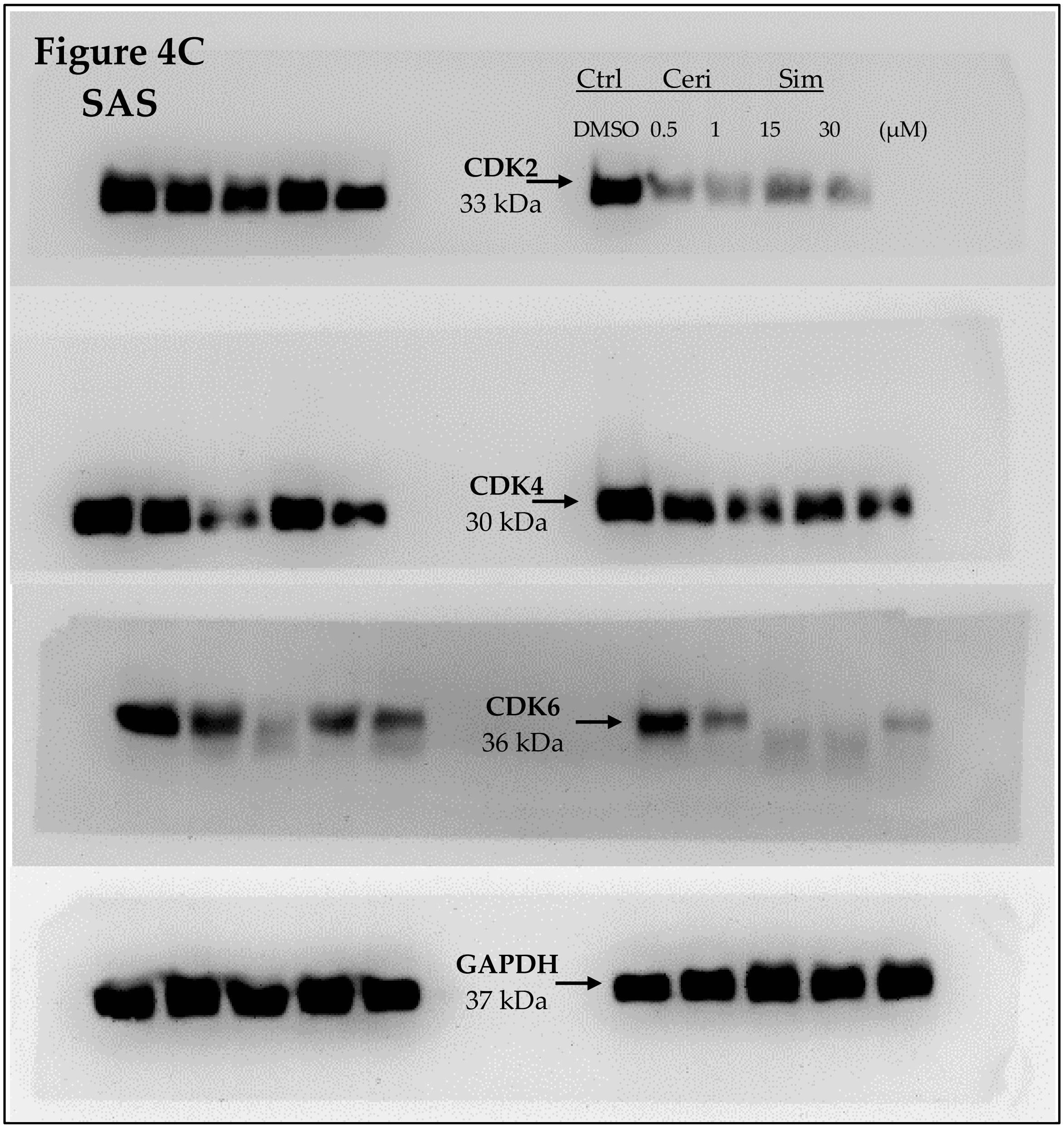

3. **DNMT1 and p21 (Figure 5)**

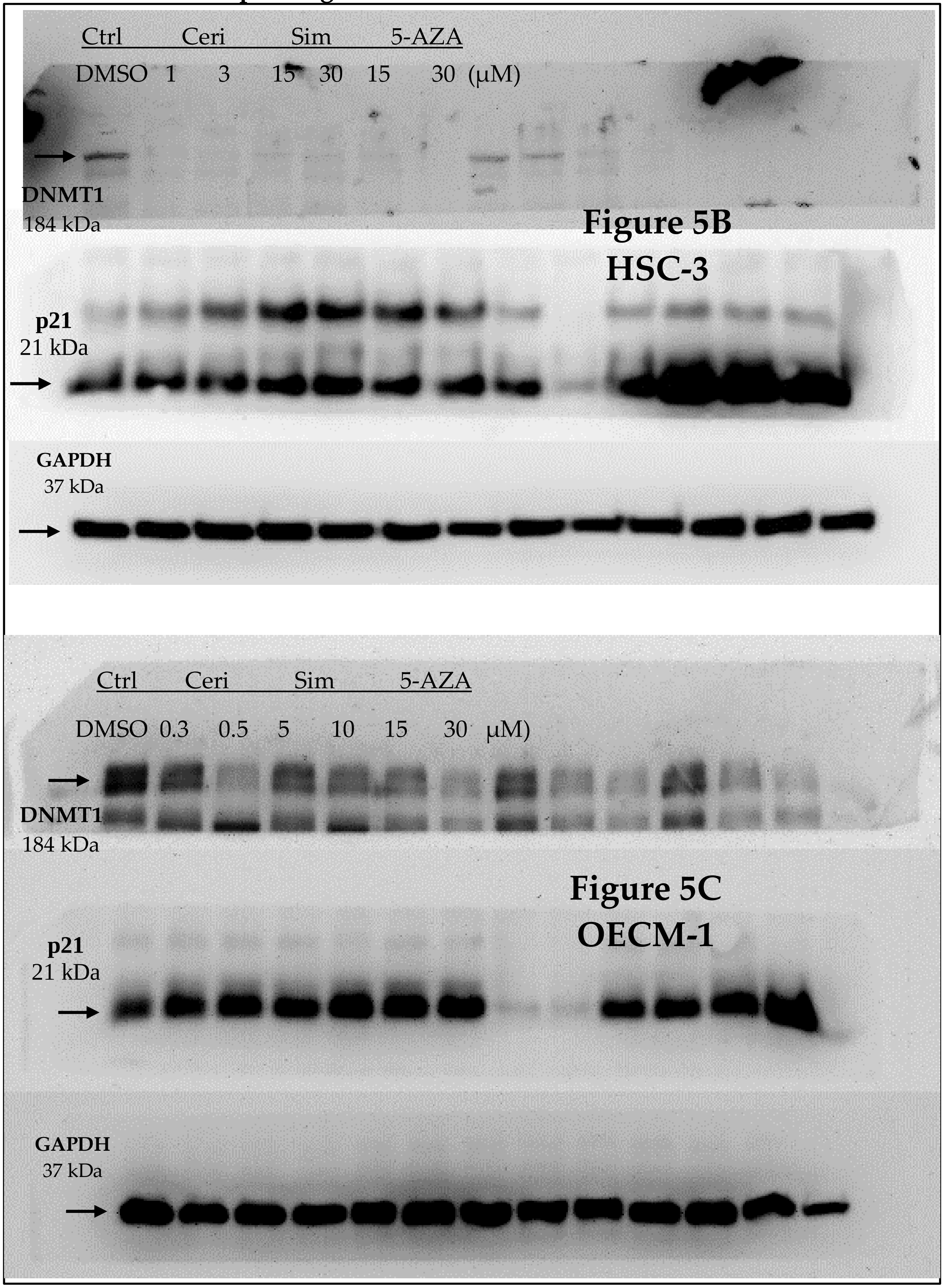

DNMT1 and p21 (Figure 5)…

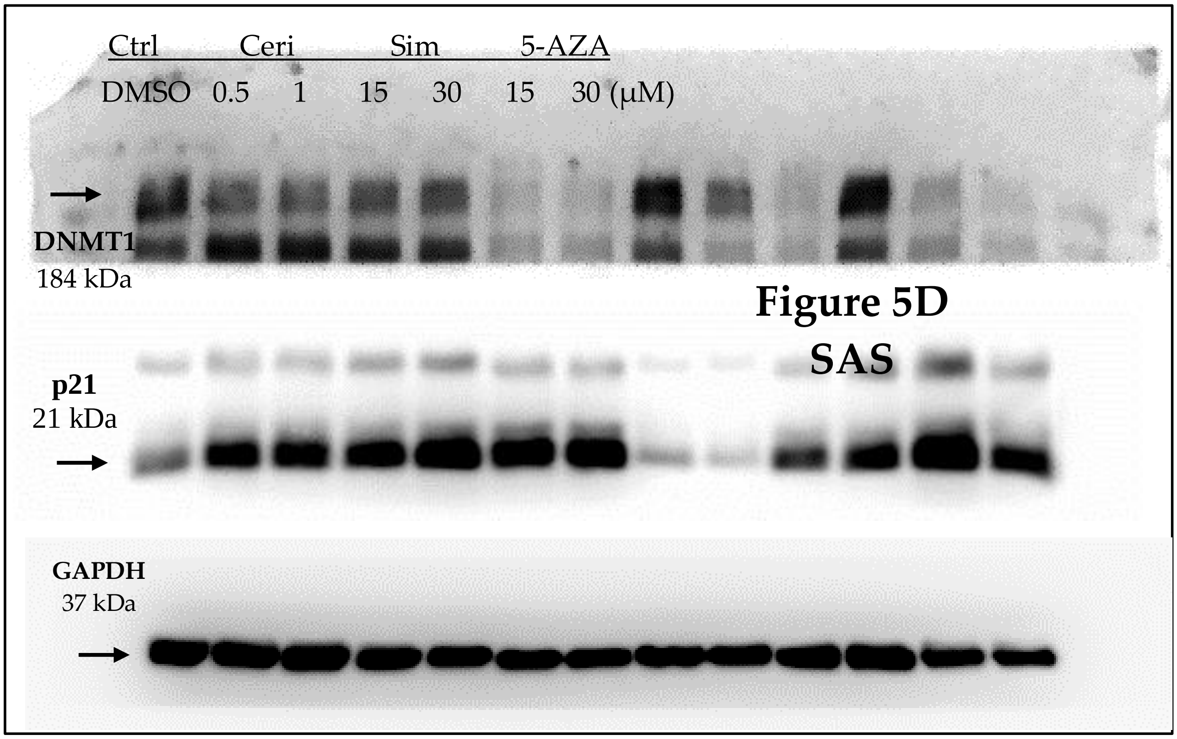

Figure S5. Original images for Western Blotting. The original images for Western blotting were shown for p21 (**A**), CDKs (**B**), and DNMT1-p21 (**C**).
